# Comorbid Premenstrual Dysphoric Disorder and Bipolar Disorder: A Review

**DOI:** 10.3389/fpsyt.2021.719241

**Published:** 2021-08-25

**Authors:** Anastasiya Slyepchenko, Luciano Minuzzi, Benicio N. Frey

**Affiliations:** ^1^Women's Health Concerns Clinic and Mood Disorders Treatment and Research Centre, St Joseph's Healthcare Hamilton, Hamilton, ON, Canada; ^2^Department of Psychiatry and Behavioural Neurosciences, McMaster University, Hamilton, ON, Canada

**Keywords:** premenstrual dysphoric disorder, bipolar disorder, women's mental health, premenstrual syndrome, comorbidities

## Abstract

Bipolar disorder (BD) differs in its clinical presentation in females compared to males. A number of clinical characteristics have been associated with BD in females: more rapid cycling and mixed features; higher number of depressive episodes; and a higher prevalence of BD type II. There is a strong link between BD and risk for postpartum mood episodes, and a substantial percentage of females with BD experience premenstrual mood worsening of varying degrees of severity. Females with premenstrual dysphoric disorder (PMDD)—the most severe form of premenstrual disturbances—comorbid with BD appear to have a more complex course of illness, including increased psychiatric comorbidities, earlier onset of BD, and greater number of mood episodes. Importantly, there may be a link between puberty and the onset of BD in females with comorbid PMDD and BD, marked by a shortened gap between the onset of BD and menarche. In terms of neurobiology, comorbid BD and PMDD may have unique structural and functional neural correlates. Treatment of BD comorbid with PMDD poses challenges, as the first line treatment of PMDD in the general population is selective serotonin reuptake inhibitors, which produce risk of treatment-emergent manic symptoms. Here, we review current literature concerning the clinical presentation, illness burden, and unique neurobiology of BD comorbid with PMDD. We additionally discuss obstacles faced in symptom tracking, and management of these comorbid disorders.

## Introduction

There is a notable impact of hormonal fluctuations on the presentation of bipolar disorder (BD), particularly during periods of reproductive life events such as pregnancy, the postpartum period, during menarche, and menopause. BD is a debilitating psychiatric disorder, characterized by episodes of mood disturbance of varying severity. BD type I is a disorder diagnosed following a fully syndromal manic episode, though most individuals with BD I also experience depressive episodes. In turn, BD type II involves the experience of at least one hypomanic episode and at least one depressive episode (1).

The prevalence and incidence of BD type I appears to be equal between sexes (2–4), though there have been some mixed findings of sex differences in prevalence and incidence of BD type II (4–6). In females, presentation of BD is marked by increased rates of rapid cycling (54.5 vs. 46.6% in one study), and the acceleration of cycles (30.3 vs. 20.3% according to one study). Female sex is linked to the worsening severity of episodes over time (50.1 vs. 39.2%) (6), as well as the presence of mixed episodes (5). Additionally, antidepressant-induced mania has been reported to occur over 3 times more frequently among females (7).

The rates of comorbidities of BD are also distinct between the sexes: male sex is associated with over two times the risk of alcohol use disorder (8, 9), and females have 0.3 times lower substance use disorder compared to males (10). Females in turn are nearly 6 more times likely to have comorbid eating disorders (10, 11), and potentially, more than double the risk of anxiety disorders, such as agoraphobia and specific phobias (11). Additionally, there is a higher likelihood of suicide attempts among females than males—one study found 39.4% of females to have a history of at least one suicide attempt, compared to 23.5% of males (6).

Interestingly, the age of onset for BD differs between males and females, with males having earlier onset of their first manic episode (12, 13). The onset of BD usually occurs between ages 15–23 in females, and the disorder usually begins with depressive, rather than manic or hypomanic episodes (14). Onset of this disorder therefore occurs during adolescence and young adulthood, meaning that the disorder overlaps with many significant reproductive life events in females, such as menarche, the perimenstrual period, the perinatal period and perimenopausal period. While the link between BD and postpartum worsening has been well-described and documented, much less is known about the link between BD and premenstrual disorders. Thus, in this review, we provide an update on studies investigating the link between BD and premenstrual disorders.

### Severe Premenstrual Syndrome and Premenstrual Dysphoric Disorder

Many females experience symptoms of affective, cognitive, and somatic disturbance during the late luteal phase of their menstrual cycle. Prior estimates have indicated that over 80% of females experience at least mild premenstrual symptoms, while ~10–21% of females experience premenstrual syndrome (PMS) (15–17). According to several estimates, premenstrual dysphoric disorder (PMDD), the most severe form of PMS that is described in the DSM-5, is experienced by 1–5% of females (17–19).

The diagnosis of PMDD is relatively new to the DSM, having been introduced as an official diagnosis for the first time in the DSM-5. To be diagnosed with PMDD, five, or more symptoms must present during the late luteal phase of the menstrual cycle (i.e., the last week of the cycle, prior to the onset of menses), and must be relieved in the week following menses. One or more of the symptoms must be a mood disturbance—that is an increase in affective lability; irritability/anger; depressed mood, hopelessness or self-deprecation; and/or increased tension, anxiety, or feeling on edge. One or more of the symptoms must also fall into the somatic or cognitive domain, including (1) a reduction in interest in activities or hobbies; (2) trouble concentrating; (3) feelings of lethargy, fatigue, or reduced energy; (4) changes in appetite; (5) sleep difficulties (i.e., insomnia or hypersomnia); (6) reports of feeling out of control, or feeling overwhelmed; (7) physical symptoms, like swelling or tenderness of breasts, reports of pain, bloating, or increased weight. Importantly, these symptoms must be tracked through prospective ratings every day for at least 2 symptomatic cycles, though a provisional diagnosis may be provided if these ratings are not available (1).

There are a number of demographic factors associated with PMDD in the community, including older age, and low socioeconomic status. PMDD is linked to a number of comorbidities, including anxiety disorders, somatoform disorders, major depressive disorder and BD (20, 21). Females with PMDD also report higher impairment and use of health and mental health services, in addition to higher suicide attempt rates (20, 22). According to prospective findings from a community-based sample from Munich, Germany, experiencing traumatic events, having a previous anxiety disorder, and daily hassles may increase the risk of developing PMDD over time (23).

Below, we provide a scoping review of the clinical presentation of comorbid BD and PMDD, and outline findings in neuroimaging and hormonal studies of biological underpinnings of these comorbid disorders. We will also discuss tracking these comorbid conditions, and potential treatment options for females with these disorders.

## Methods

To conduct this scoping review, we searched the PubMed/Medline database using an inclusive search strategy with the keywords “bipolar” or “manic depressive” combined with “premenstrual” or “late luteal,” limiting our search to articles published in the English language up until April 2021, yielding a total of 148 items. We selected articles which described premenstrual exacerbation, premenstrual symptoms of any severity, and PMDD among individuals with BD. We focused our review on clinical characteristics, biological underpinnings, neurobiology, symptom tracking and treatment of premenstrual symptoms of any severity in BD. A summary of the findings of our review, describing differences in individuals with BD comorbid with premenstrual symptoms of different severities can be found in Table 1.

**Table 1:** Clinical and neurobiological characteristics associated with premenstrual exacerbation, severe premenstrual symptoms, and premenstrual dysphoric disorder in bipolar disorder.

**Clinical and Neurobiological Characteristic**	**Premenstrual Symptom Comorbidity**	**BD with Premenstrual Symptom Comorbidity Compared to BD Only**
Mood episodes	BD-PME	Increased number of depressive episodes (24)
	BD-Severe PMS	Not linked to number of episodes or episode duration (25)
	BD-PMDD	More [hypo]manic or depressive episodes in past year, more lifetime depressive episodes (26)
Symptom severity: depression and mania	BD-PME	Higher symptom severity for manic and depressive symptoms (24)
	BD-PMDD	**Follicular:** worsened depressive symptoms, no differences in manic symptoms.**Late luteal**: similar depressive symptoms, worse manic symptoms (27)
Symptom severity: anxiety	BD-PMDD	**Late luteal:** higher state anxiety. No differences in trait anxiety (27)
Biological rhythms disruption and sleep quality	BD-PMDD	**Follicular and late luteal**: worsened subjective biological rhythms disruptions, similar sleep quality (27)
Rapid cycling	BD-PMDD	Mixed findings-higher risk of rapid cycling (26), no link to rapid cycling (28)
Relapse	BD-PME	Linked to shorter time to relapse of syndromal/subsyndromal mood episode (24)
Cyclothymia	BD-PMDD	Associated with cyclothymia (28).
Bipolar disorder type	BD-PMDD	Mixed findings: associated with BD type II (28, 29), not associated with BD type I or II (26)
First episode polarity	BD-PMDD	Not linked to first episode polarity (26)
Age of onset	BD-Severe PMS	Linked to earlier age of onset (25).
	BD-PMDD	Mixed findings. Linked to earlier onset of depressive and manic episodes (26), not linked to age of onset (28).
Seasonal pattern	BD-PMDD	Not linked to seasonal pattern (28).
Psychotic features/past hospitalization	BD-PMDD	Not linked to psychotic features/past hospitalization (28).
Comorbid conditions	BD-PMDD	Linked to history of anxiety disorder (including generalized anxiety disorder, panic disorder with and without agoraphobia), lifetime substance/ alcohol abuse or dependence, lifetime bulimia nervosa, lifetime post-traumatic stress disorder and adult attention deficit and hyperactivity disorder (26), body dysmorphic disorders (28). Mixed findings: BD-PMDD linked to obsessive compulsive disorder according to Fornaro and Perugi (28), not linked to obsessive compulsive disorder according to Slyepchenko et al. (26).
Oral contraceptives	BD-PMDD	Linked to more severe mood symptoms during oral contraceptive use (26)
Reproductive life events: menarche, pregnancy, postpartum, menopause	BD-PME	Mood worsening in perimenstrual, postnatal, menopausal period (30)
	BD-Severe PMS	No link between mood symptoms during premenstrual period with postnatal or perimenopausal period in BD type I (31).
	BD-PMDD	Linked to earlier age at menarche and shorter span of time between menarche and BD onset (26), linked to mood symptoms during pregnancy and postpartum (26, 28).
Hormones	BD-PMDD	No difference in hormone levels (β17-estradiol, progesterone, allopregnanolone, dehydroepiandosterone sulfate) (27)
Resting-state functional connectivity (magnetic resonance imaging)	BD-PMDD	Higher functional connectivity between L hippocampus and R frontal cortex Decreased functional connectivity between R hippocampus and R premotor cortex (27).
Cortical thickness	BD-PMDD	Decreased cortical thickness in L superior frontal, L. pericalcarine, L. superior parietal gyri, R middle temporal and rostral middle frontal cortices. Increased cortical thickness in L superior temporal gyrus (27).
Subcortical structure volume	BD-PMDD	**Late luteal** – increased volume in L caudate nucleus. No differences in R caudate, bilateral putamen, hippocampus, amygdala, thalamus, accumbens area, ventral encephalon (27)

*BD, bipolar disorder; BD-PMDD, bipolar disorder comorbid with premenstrual dysphoric disorder; L, left; PMDD, premenstrual dysphoric disorder; PME, premenstrual exacerbation; PMS, premenstrual syndrome; R, right*.

## Severe Premenstrual Syndrome and Premenstrual Dysphoric Disorder in Bipolar Disorder

Among females with BD, 45 to 68% report having premenstrual mood symptoms varying in severity (2, 26, 29, 31, 32). Similarly, females with PMDD are much more likely to have a diagnosis of BD compared to the general population, with an 8-fold increase in risk reported by one study (20), and 2.3 times higher prevalence of BD reported by a study among young females (21). Persons who have comorbid PMDD and BD have been reported to have a worse course of illness, marked by a higher number of episodes, higher comorbidities, higher rates of rapid cycling, and other factors that amount to worse clinical outcomes (26).

## Clinical Presentation of Comorbid Bipolar Disorder and Premenstrual Dysphoric Disorder

There have been a number of studies investigating the course of illness and clinical presentation in females with premenstrual exacerbation of mood symptoms or PMDD comorbid with BD, showing worsened clinical presentation of BD in these comorbid disorders, including a higher number of episodes, shorter time to relapse, earlier age of onset, risk of rapid cycling, and higher number of comorbidities (24, 26).

In a prospective study of 293 females with BD from the Systematic Treatment Enhancement Program for BD (STEP-BD), Dias and colleagues found that females with premenstrual exacerbation of mood symptoms had a higher number of depressive episodes, had a shorter time to relapse to a subsyndromal or syndromal episode of BD (4.5 months compared to 8.5 months). Additionally, symptom severity among these females was higher for both depressive and manic symptoms (24). In a cross-sectional study of the STEP-BD data, Slyepchenko et al. defined a provisional PMDD diagnosis in females enrolled in STEP-BD based on DSM-5 criteria. Having PMDD was linked to a higher risk of rapid cycling, as well as [hypo]manic and depressive episodes in the past year, and lifetime depressive episodes. Findings from this study also showed that in females with BD, having PMDD was not associated with BDI or BDII, or first episode polarity (26). However, in a smaller study by Fornaro and Perugi (*n* = 92 females with BD, 25 of whom had PMDD), having PMDD was linked to cyclothymia and BD-II, though not rapid cycling, any association with age of onset, a seasonal pattern, or psychotic features/ past hospitalization (28). Another small study did not find an association of number of depressive or manic episode, or length of depressive episodes to be linked to severe premenstrual mood symptoms (25). Additionally, several smaller studies have suggested the association of severe PMS and PMDD with BD-II, though this finding was not confirmed in a larger sample (26, 28, 29). Earlier age of onset of BD in females with both BD and PMDD/ premenstrual mood symptoms has been reported by two studies, one of which reported earlier onset of both [hypo]manic and depressive episodes (25, 26).

**Figure 1:**
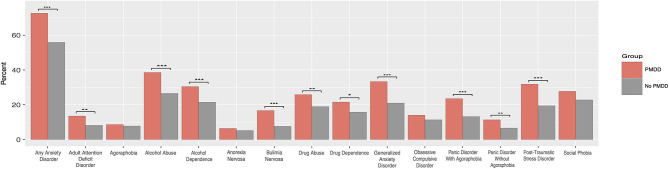
Psychiatric comorbidities in women with bipolar disorder with and without comorbid premenstrual dysphoric disorder. ^***^p < 0.001; ^**^p < 0.01, ^*^p < 0.05. PMDD, Premenstrual Dysphoric Disorder; OCD, Obsessive Compulsive Disorder. Reproduced with permission from Slyepchenko et al. (26).

Females with comorbid PMDD and BD are affected by a higher rate of comorbid conditions (see Figure 1), including having a history of anxiety disorders, such as generalized anxiety disorder, panic disorder with and without agoraphobia. Additionally, they are more likely to have a lifetime history of substance / alcohol abuse or dependence, and a lifetime history of bulimia nervosa. As well, individuals with the comorbid disorders are more likely to have a history of post-traumatic stress disorder (PTSD) and adult attention deficit and hyperactivity disorder (ADHD) (26). In another study, PMDD was linked to obsessive compulsive disorder (OCD) and Body Dysmorphic Disorders as well (28), though the link to OCD was not replicated by a larger study (26).

Individuals with comorbid BD and PMDD also have an earlier age of menarche, as well as a shorter span of time between the onset of menarche and the onset of BD. Additionally, they were found to be more likely to have severe mood symptoms during oral contraceptive use. These findings indicate that hormonal changes may play a role in these comorbid conditions (26). To further support this, several reports indicate that individuals with both BD and PMDD are more likely to have mood symptoms during other reproductive life events, such as pregnancy and the postpartum period (26, 28), with one study reporting mood worsening in 77% of women during the perimenstrual, postnatal or menopausal periods, which were linked to a worsened course of BD, including earlier age of onset (30). However, these findings have not been consistent: Payne and colleagues reported no association between mood symptoms during different reproductive life events (i.e., premenstrual period, postpartum, perimenopausal) in women with BD type I (31).

In a study from our group where all participants completed at least 2 months of prospective daily symptom charting, we found that individuals diagnosed with both BD and PMDD had worse depressive symptom severity during the follicular phase compared to females with BD, females with PMDD, a female control group, and individuals with both BD and PMDD. Additionally, females with BD and PMDD had worsened depressive symptoms during the late luteal phase compared to controls, but not the other groups—individuals with a PMDD diagnosis were equally symptomatic. In terms of manic symptoms, females in the BD-PMDD group had worsened manic symptoms during the late luteal phase compared to all other groups, though these differences were not present during the follicular phase of the menstrual cycle. Additionally, females with BD-PMDD had higher state anxiety during the follicular phase compared to the control group during the follicular phase, and higher state anxiety compared to both the BD and control groups by the late luteal phase. They also had higher trait anxiety compared to the PMDD group and the control group during the follicular phase, though trait anxiety scores worsened for females with PMDD only by the luteal phase (27). Additionally, females with both BD and PMDD had worsened subjective biological rhythms disruptions than those with BD, PMDD, and healthy controls during the follicular phase. However, by the luteal phase, females in the PMDD group had no statistical differences in subjective biological rhythms disruption compared to females with both BD and PMDD. Notably, females with both BD and PMDD had worse subjective sleep quality during the luteal phase than both the PMDD group and control groups, though not the BD-only group. Overall, these findings indicate that symptomatic burden is worsened in individuals euthymic for BD, with comorbid PMDD, in terms of depressive symptoms, anxiety symptoms, sleep and biological rhythms disruption, with some differential effects based on menstrual phase. It is possible that increased symptoms during euthymia may be a contributing factor to the increased frequency of depressive and manic episodes seen in comorbid BD/ PMDD (27).

## Biological Underpinnings of Premenstrual Dysphoric Disorder

The physiological control of the menstrual cycle in females is regulated by the hypothalamic-pituitary-gonadal axis. The hypothalamic-pituitary-gonadal axis itself is composed of a number of feedback mechanisms, where the hypothalamus releases gonadotropin-releasing hormone (GnRH), which travels to the pituitary through the hypophyseal portal veins and stimulates the anterior pituitary gland to produce luteinizing hormone (LH) and follicle-stimulating hormone (FSH). In the follicular phase—the first portion of the menstrual cycle—FSH acts on the ovary to grow follicles, which at the end of the follicular phase begins to secrete high levels of estradiol. The estradiol creates a positive feedback effect on the pituitary's release of luteinizing hormone, which leads to ovulation—the release of an oocyte from the mature follicle. The follicle now becomes the corpus luteum, which begins to secrete progesterone and estradiol, in the process of luteinization, as induced by LH, comprising the luteal phase. The high levels of estrogen and progesterone feedback to FSH and LH secretion, leading the diminishment in the levels of these hormones. During the late luteal phase, the corpus luteum becomes inactive, resulting in a drop in levels of progesterone and estradiol at the end of the luteal phase, which leads the uterine lining to shed, and menses to commence (33).

As symptoms of PMDD occur predictably in accordance with the menstrual phase, some studies of the biological underpinnings of premenstrual syndrome and its severe counterparts investigated levels of circulating steroids and gonadotropins in women with PMDD. These studies hypothesized that abnormal levels of circulating steroids and gonadotropins were the cause of this disorder. However, these studies did not find differences in hormones such as plasma estradiol, progesterone, testosterone, FSH, or LH levels to differ in PMDD compared to controls (34, 35).

In a series of elegant studies, Schmidt and Rubinow used the progesterone receptor antagonist mifepristone (also known as RU-486), which is able to initiate menses and terminate activity of the corpus luteum, with or without simultaneous administration of human chorionic gonadotropin (hCG), which is able to maintain the activity of the corpus luteum during the administration of mifepristone, while continuing the effects of the progesterone antagonist and while initiating menses. This method allows to maintain the presence and effects of a luteal phase, or eliminate them, while initiating menses in a blinded fashion. Females receiving mifepristone with or without hCG continued to express symptoms of PMDD in the follicular phase, indicating that the luteal phase is not mandatory for PMDD to be present (36).

Schmidt et al. then demonstrated that eliminating the menstrual cycle and associated ovarian suppression through a GnRH agonist (leuprolide) led to the elimination of symptoms of PMDD in a majority of the females—findings that were confirmed by a later meta-analysis, showing that GnRH agonists are effective at reducing premenstrual symptoms (37, 38). Adding back estradiol and progesterone in these females led to a return of PMDD symptoms. However, when this procedure was performed in women with no history of PMDD, no effects on mood were seen, demonstrating the presence of an effect of circulating progesterone and estradiol on mood specifically in individuals who are sensitive to the hormonal changes (37). Similarly, converging evidence suggests that suppression of ovulation that occurs naturally or through hysterectomy and oophorectomy, combined with continuous estrogen replacement leads to the amelioration of PMDD symptoms in most females (39, 40).

Schmidt and colleagues investigated whether it was the change in steroid levels that appeared to initiate the onset of PMDD symptoms, by administering 3 months of a continuous level of estradiol and progesterone to responders to the GnRH agonists. Symptoms of PMDD were only increased during the first month of the add-back of the combined estradiol and progesterone treatment, followed by stable mood for the following 2 months, indicating that it was indeed changes of estradiol and progesterone that were associated with PMDD symptoms, rather than increased stable levels of the hormones (41).

The mechanisms responsible for these effects, however, remain unknown. One major hypothesized mechanism behind PMDD is the activity of neurosteroid metabolites of progesterone, such as allopregnanolone (3α-hydroxy-5α-pregnan-20-one). Allopregnanolone is metabolized from progesterone in the brain and in the corpus luteum (42, 43), and importantly, is a positive allosteric modulator for gamma-aminobutyric acid (GABA)-A receptors (44–46). This metabolite rises and falls accompanying the levels of progesterone during the menstrual cycle (47). It is able to produce anxiolytic (48, 49), sedative (50), and anti-seizure effects (51). It appears that there is a paradoxical effect of allopregnanolone in some individuals, and at high levels, this neurosteroid may induce negative mood effects (52). Interestingly, treatment of PMDD with dutasteride, a 5α-reductase inhibitor which blocks the conversion of progesterone into allopregnanolone, reduces PMDD symptoms during the late luteal phase, including sadness, irritability, food cravings, bloating, and anxiety (53). Animal models also suggest that changes in progesterone levels and their metabolites can lead to altered conformation of GABA-A receptors. In turn, stabilizing levels of hormones normalize receptor conformation (54–56).

On a cellular level, Schmidt and Rubinow's group found in that in ovarian steroid-free lymphoblastoid cell line cultures from females with PMDD responsive to GnRH agonists, one of the most significantly over-expressed pathways was the Extra Sex Combs/ Enhancer of Zester (ESC/E(Z)) complex. Moreover, the lymphoblastoid cell line cultures in PMDD have a different sensitivity to estrogen/progesterone compared to equivalent cultures from women without premenstrual mood symptoms, reflected in ESC/E(Z) complex expression: in control cell lines, expression of some ESC/E(Z) genes were increased by progesterone, but in PMDD cell lines, they were decreased by estradiol (57). The effects of ovarian steroids may therefore be differentially regulated through epigenetic pathways in females with and without PMDD.

Another potential underlying biological component of PMDD is the effects seen in the brains of females with PMDD through functional and structural imaging. For instance, studies have identified a number of candidate regions for differential activity of steroids. In GnRH agonist-induced ovarian suppression in women with PMDD, combined with estradiol and progesterone add-back, women with PMDD exhibited increased activation in the bilateral dorsolateral prefrontal cortex, medial frontal gyrus and the cerebellum (58).

## Neurobiology of Comorbid Premenstrual Dysphoric Disorder and Bipolar Disorder

The neurobiological underpinnings of PMDD comorbid with BD have been little-investigated to date. However, there are some emerging findings related to hormone levels, structural and functional neuroimaging beginning to understand the comorbidity of these disorders.

To our knowledge, no study to date has investigated the genetic underpinnings of comorbid BD and PMDD. Payne and colleagues did not find severe premenstrual mood symptoms in BD-I to exhibit familiality (25).

Our group investigated structural and functional magnetic resonance imaging (MRI), as well as serum levels of several hormones and metabolites in 19 females with BD comorbid with PMDD compared to a female control group (*n* = 25), 20 females with BD, and 25 females with PMDD during the mid-follicular and late luteal phases of their menstrual cycle. Participants enrolled in the study were euthymic for BD symptoms for at least 2 months prior to enrolling in this study (27). No differences in hormone levels were found for either the mid-follicular or the late luteal phase of the menstrual cycle for the control group, individuals with BD, or BD comorbid with PMDD, for levels of β17-estradiol, progesterone, allopregnanolone, or dehydroepiandosterone sulfate. Notably, levels of allopregnanolone were elevated in individuals with PMDD only, during both the follicular and late luteal phases (27).

**Figure 2:**
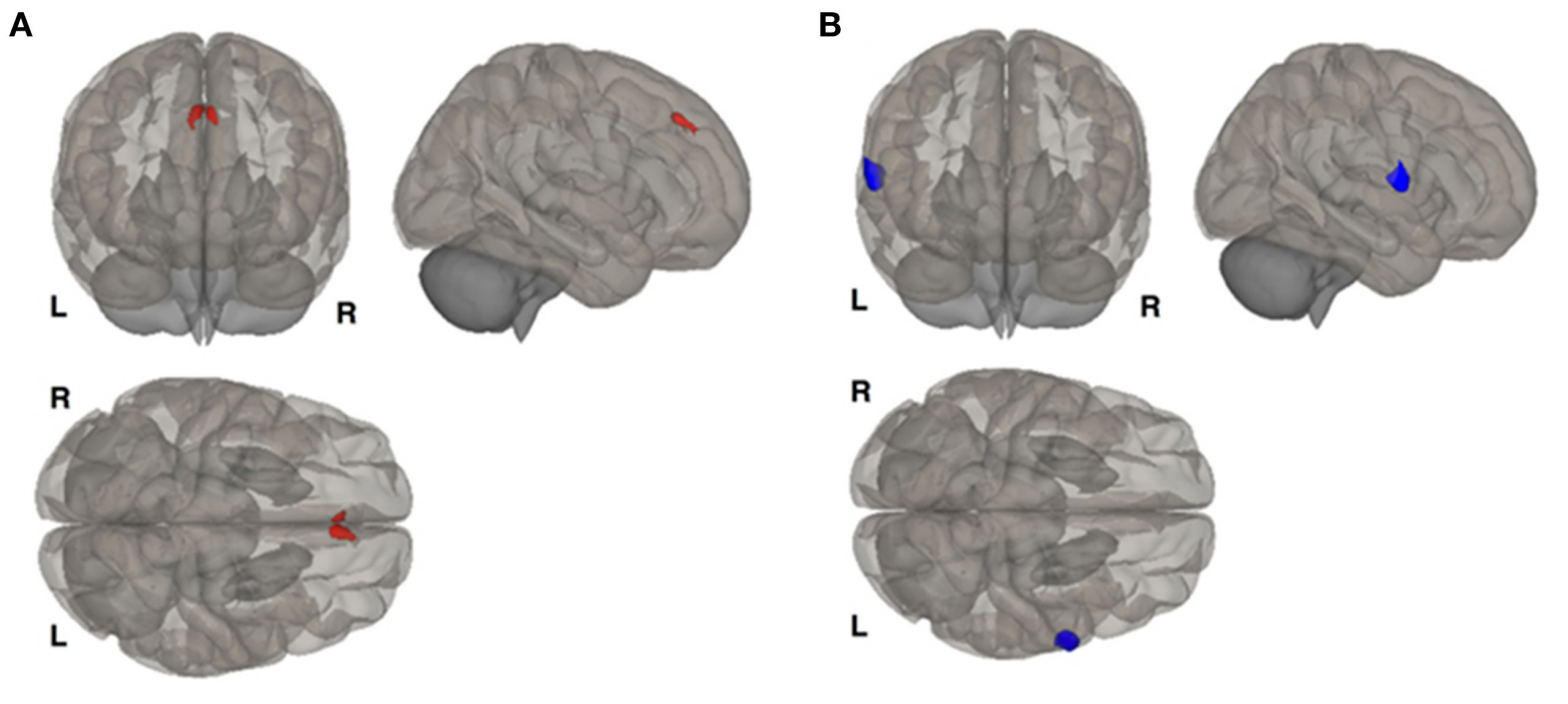
Resting state functional connectivity differences between females with bipolar disorder and females with bipolar disorder and comorbid premenstrual dysphoric disorder. **(A)** Shows that left hippocampus seed is more connected with the right frontal cortex in females with the comorbid disorders. **(B)** Shows that right hippocampus is less functionally connected with left premotor cortex in females with the comorbid disorders. Results corrected for multiple comparisons using false discovery rate procedure. Reproduced with permission from Syan et al. (27).

As we investigated whole-brain resting-state functional connectivity (Figure 2), we found differences in functional connectivity between the right hippocampus and right premotor cortex in females with BD and PMDD in a seed-based approach. During the late luteal phase, in a seed-to-voxel-based analysis of resting-state functional connectivity MRI, with seeds based in the bilateral hippocampus, our research group found increased functional connectivity between the left hippocampus and right frontal cortex and decreased functional connectivity between the right hippocampus and the right premotor cortex in females with both BD and PMDD, compared to females with BD only. Females with BD and PMDD had increased functional connectivity between the right hippocampus and left frontal cortex, bilateral dorsolateral prefrontal cortex, and decreased functional connectivity between the right hippocampus and bilateral primary motor cortex, and left hippocampus with left somatosensory cortex compared to females with PMDD only. These findings revealed that specific fronto-limbic pathways are part of the underlying mechanisms involved in comorbid BD and PMDD (27). Notably, the findings of decreased coupling between functional connectivity between the hippocampus with somatosensory and motor areas are similar to those found in bipolar depression (27, 59). When using the bilateral ventrolateral prefrontal cortex, ventromedial prefrontal cortex, amygdala, or postcentral gyrus as seed points, no differences were found between groups. It is important to note that estrogen receptor β mRNA expression is high in the hippocampus, giving a putative role for hormonal influence on these networks (27, 60).

**Figure 3:**
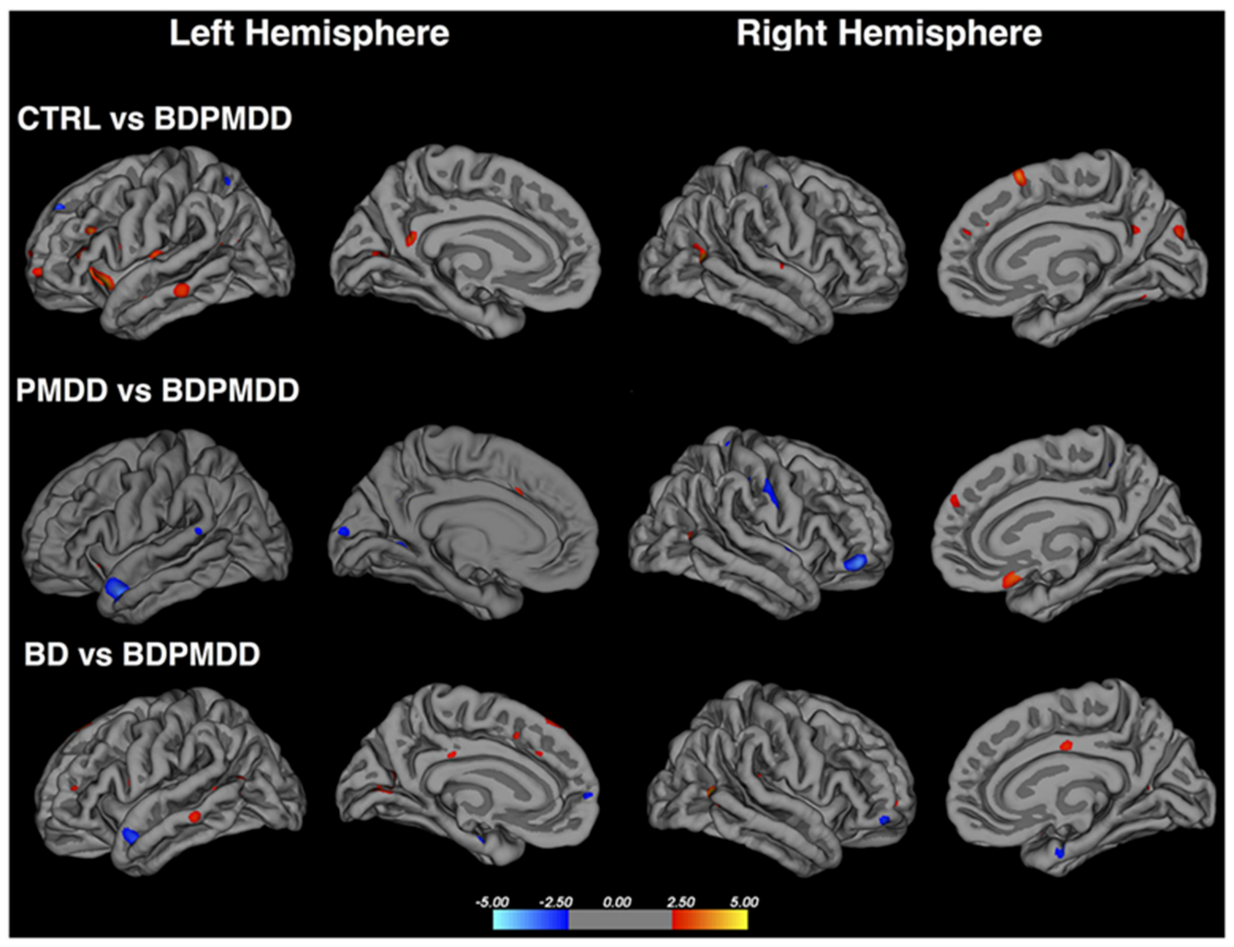
Cortical thickness differences in females with both bipolar disorder and premenstrual dysphoric disorder to a control population, females with premenstrual dysphoric disorder only and females with bipolar disorder only. Red regions indicate higher cortical thickness in the first group compared to females with bipolar disorder and premenstrual dysphoric disorder, while blue regions indicate lower cortical thickness in the first group compared to females with bipolar disorder and premenstrual dysphoric disorder. BD, bipolar disorder; BDPMDD, bipolar disorder with comorbid premenstrual dysphoric disorder; CTRL, healthy controls; PMDD, premenstrual dysphoric disorder. Reproduced with permission from Syan et al. (27).

This study additionally investigated cortical thickness between groups, finding cortical thinning in females with comorbid BD and PMDD, as compared to BD in a number of areas (Figure 3). These included the left superior frontal, left pericalcarine, left superior parietal gyri, and the right middle temporal, and rostral middle frontal cortices. Cortical thickness increases were seen in the left superior temporal gyrus. Compared to individuals with PMDD only, those with BD and PMDD displayed cortical thinning in the right medial orbitofrontal cortex, right inferior parietal cortex, and thickening in the left superior temporal cortex, right pars orbitalis, left lingual and right superior parietal gyri. Finally, compared to females without any psychiatric diagnoses, individuals with both BD and PMDD had cortical thinning in the insula, right middle temporal gyrus, right medial orbitofrontal cortex, left pars triangularis, left rostral middle frontal cortex, right cuneus, and right superior frontal cortex. Overall, these findings indicate the cortex of females with BD and PMDD is particularly impacted by these comorbid disorders (27).

Additionally, there was increased volume in the left caudate nucleus in females with BD-PMDD compared to those with BD only during the late luteal phase of the menstrual cycle. However, no differences were found in the right caudate, bilateral putamen, hippocampus, amygdala, thalamus, accumbens area, or ventral encephalon (27). Overall, females with both BD and PMDD appear to experience a unique impact from these disorders on their brain structure and function, as compared to females with BD or PMDD only, and those without any psychiatric disorders. Findings of the unique structural and functional impact of comorbid BD and PMDD underscore the idea that the comorbidity of these disorders is linked to a worsened course of illness in BD (27).

## Symptom Tracking in Comorbid Bipolar Disorder and Premenstrual Dysphoric Disorder

Special attention should be paid to symptom tracking in comorbid BD and PMDD. Given that both BD and PMDD are characterized by changes in overlapping symptoms (e.g., irritability, low mood, somatic symptoms), the use of prospective, daily symptom tracking tools is essential to accurately establishing a diagnosis of PMDD in individuals with BD. To confirm a diagnosis of PMDD, symptoms must be tracked for a period of a minimum of two symptomatic cycles (1). The most commonly used clinical tool used to track premenstrual symptom severity is the Daily Record of Severity of Problems (61). However, this tool does not concurrently track symptom severity of manic and depressive symptoms. To address this, the McMaster Premenstrual and Mood Symptom Scale (MAC-PMSS) was developed to prospectively track manic and depressive symptoms simultaneously with premenstrual symptoms, in addition to menstrual bleeding, in order to provide an accurate understanding of the clinical picture, and to be able to accurately provide a diagnosis of PMDD in females with BD (62).

Ideally, a diagnosis of PMDD in females with BD should be confirmed while their BD is relatively stable, and individuals are currently not in an acute depressive or [hypo]manic episode (63), in order to ensure appropriate treatment and diagnosis. Daily symptom tracking should be performed once symptoms of BD are stabilized, in order to be able to confirm that the symptoms that occur during the perimenstrual period are not secondary to BD (63). To perform a differential diagnosis, symptoms specific to diagnostic criteria of PMDD should be tracked throughout the course of at least two menstrual cycles, in addition to menstrual bleeding and spotting, as well as depressive and manic symptoms. Clinicians can then use the prospective charting to verify whether there is cyclic change in premenstrual symptoms that is attributable to the late luteal phase, as opposed to change in depressive and manic symptoms unrelated to the premenstrual cycle phase.

## Treatment Considerations of Comorbid Premenstrual Dysphoric Disorder in Bipolar Disorder

Treatment of severe PMS and PMDD in females with BD is a challenging and little-investigated topic. There have been several case studies describing treatment in this population, however no randomized, placebo-controlled trials have investigated treatment of PMDD when comorbid with BD. However, these case studies and the literature available on treatment of PMDD in the general population, as well as careful consideration of its interactions with treatment for BD can begin to guide future investigations of this under-researched topic.

The first-line treatment of severe PMS and PMDD in the general population is treatment with selective serotonin reuptake inhibitors (SSRIs) either continuously, or taken during the luteal phase (64–66). However, treatment of PMDD with SSRIs in females with BD poses additional concerns due to risk of antidepressant-induced mania (67). Moreover, if patients are clinically stable for BD when their diagnosis of PMDD is established, prescribers may be hesitant to prescribe antidepressants and risk potential destabilization and mood worsening (63). Another available intervention for severe PMS and PMDD is the use of combined oral contraceptives, which have been shown to decrease premenstrual symptoms and the associated functional impairment in individuals with these disorders (68). Additionally, there are a number of non-pharmacological interventions that have been shown to be effective in treating premenstrual symptoms, including cognitive behavioral therapy (69), exercise (70), and supplementation with vitex agnus castus (71), although none of these treatments have been reported in individuals with co-morbid BD and PMDD. Finally, more radical treatment of PMS/PMDD may involve treatment with GnRH analogs and adding back estrogen and progesterone therapies, or even surgical interventions (66, 72).

Case studies from Frey and Minuzzi (73) and Smith and Frey (63) described successful instances of treatment of PMDD in BD using adjunctive treatment with hormonal contraceptives, specifically, a continuous transdermal patch of norelgestromin 6.0 mg and ethinyl estradiol 0.60 mg; or combined oral contraceptives containing either 3.0 mg drospirenone and 0.030 mg of ethinyl estradiol or 0.15 mg levonorgestrel and 0.020 mg of ethinyl estradiol (63, 73). It should be noted, that in one case, the combined oral contraceptive was discontinued due to emergent headaches and weight gain, in spite of mood improvement (73). However, given that these are the only cases available of management of PMDD in BD, these case studies are promising, and interventions using hormonal contraceptives for treatment of PMDD should be tested in randomized, controlled trials.

In the absence of robust clinical trials in women with comorbid BD and PMDD, Smith and Frey provided some clinical recommendations for the management of PMDD in BD. They recommend psycho-education; lifestyle interventions that target diet, activity and sleep hygiene; calcium and vitamin B6 supplementation; cognitive behavioral therapy interventions; hormonal intervention and, in cases of severe PMDD that failed use of hormonal interventions, treatment with adjunctive antidepressants may be needed (63). Clinicians need to be aware some individuals with BD may feel worse when using antidepressants or oral contraceptives (e.g., more depressed, anxious, irritable, agitated, or even suicidal), so close clinical monitoring is very important in this population. Studies investigating the treatment of PMDD among individuals with BD remain as one of the major research gaps, and an unmet clinical need for these comorbid conditions.

## Conclusions

Severe premenstrual symptoms and PMDD are linked to high clinical severity, worsened symptomatology and a severe course of illness in individuals with BD. This increased illness burden is accompanied by underlying structural and functional cortical abnormalities, which are likely either an underlying mechanism or reflection of the increased clinical burden in this population. However, in spite of this increased clinical burden, there are few studies that have investigated the neurobiological underpinnings of the comorbid disorders, and especially, treatment and management of PMDD in BD. There is a lack of clinical trials that have evaluated any pharmacological, psychotherapeutic, or adjunctive treatment in PMDD among individuals with BD. Treatment of PMDD in individuals with BD is more complex, due to the possibility of antidepressant-induced mania, and overall, maintaining clinical stability across both disorders. Tracking symptoms of PMDD at the same time as manic and depressive symptoms, in addition to stabilizing BD prior to tracking symptoms of PMDD, are critical clinical steps that can help clinicians and researchers to ensure an accurate diagnosis. Future prospective investigations of the course of illness and its neurobiology will allow for a more accurate picture of disease progression in these comorbid disorders.

## Author Contributions

AS: conceptualized review and wrote first draft of manuscript. BF and LM: conceptualized review and wrote manuscript. All authors contributed to the article and approved the submitted version.

## Conflict of Interest

The authors declare that the research was conducted in the absence of any commercial or financial relationships that could be construed as a potential conflict of interest.

## Publisher's Note

All claims expressed in this article are solely those of the authors and do not necessarily represent those of their affiliated organizations, or those of the publisher, the editors and the reviewers. Any product that may be evaluated in this article, or claim that may be made by its manufacturer, is not guaranteed or endorsed by the publisher.
